# Improving stigma and psychosocial outcomes among post-abortion Kenyan women attending private clinics: A randomized controlled trial of a person-centered mobile phone-based intervention

**DOI:** 10.1371/journal.pone.0270637

**Published:** 2022-06-24

**Authors:** May Sudhinaraset, Amanda Landrian, Sun Yu Cotter, Ginger Golub, James Opot, C. Avery Seefeld, Beth Phillips, Edward Ikiugu

**Affiliations:** 1 Fielding School of Public Health, University of California, Los Angeles, Los Angeles, California, United States of America; 2 Institute for Global Health Sciences, University of California, San Francisco, San Francisco, California, United States of America; 3 Innovations for Poverty Action, Nairobi, Kenya; 4 Marie Stopes Kenya, Nairobi, Kenya; PLOS (Public Library of Science), UNITED KINGDOM

## Abstract

**Objectives:**

The objective of this study was to evaluate a person-centered abortion care mobile-based intervention on perceived social stigma, social support, mental health and post-abortion care experiences among Kenyan women who received abortion services at private clinics.

**Methods:**

This randomized controlled study enrolled women who obtained an abortion from private clinics in Nairobi county, Kenya and randomized them into one of three study arms: 1) standard of care (follow-up by service provider call center); 2) post-abortion phone follow-up by a peer counselor (a woman who has had an abortion herself and is trained in person-centered abortion care); or 3) post-abortion phone follow-up by a nurse (a nurse who is trained in person-centered abortion care). All participants were followed-up at two- and four-weeks post-abortion to evaluate intervention effects on mental health, social support, and abortion-related stigma scores. A Kruskal-Wallis one-way ANOVA test was used to assess the effect of each intervention compared to the control group. In total, 371 women participated at baseline and were each randomized to the study arms.

**Results:**

Using Kruskal-Wallis tests, the nurse arm improved mental health scores from baseline to week two; however this was only marginally significant (p = 0.059). The nurse arm also lowered stigma scores from baseline to week four, and this was marginally significant (p = 0.099). No other differences were found between the study arms. This person-centered mobile phone-based intervention may improve mental health and decrease perceived stigma among Kenyan women who received abortion services in private clinics.

**Conclusions:**

Nurses trained in person-centered abortion care, in particular, may improve women’s experiences post-abortion and potentially reduce feelings of shame and stigma and improve mental health in this context.

## Introduction

In Kenya, women’s access to safe abortion services remains highly restricted despite legal reforms coupled with efforts to improve accessibility. Abortions are permitted only if a trained health professional deems the woman’s life or health is threatened; however, the ambiguous interpretation contributes to challenges in obtaining safe abortions [1]. Approximately 464,000 Kenyan women had abortions in 2012 [2] and 2,600 women die annually from unsafe abortions [3]. Efforts are needed to improve women’s abortion care.

Studies have identified abortion stigma as a primary barrier for Kenyan women [4], defined as a “negative attribute ascribed to women who seek to terminate a pregnancy that marks them, internally or externally, as inferior to the ideals of womanhood,” resulting in social isolation and secrecy [5]. In Kenya, abortion is associated with accusations of promiscuity or engaging in transactional sex work [4]. One study found that reducing stigma may reduce post-abortion psychological distress [6], while another finds that social support from partners and trusted individuals reduced perceived stigma [7]. Despite the pervasiveness of stigma and potential health consequences, there have been few interventions focused on stigma reduction, particularly in highly restrictive settings like Kenya.

Due to its sensitivity, several studies call for confidential social support interventions and accurate informational support through innovative mobile health (mHealth) approaches [4, 8]. One systematic review identified health professionals and peer counselors as instrumental to counter abortion stigma—two studies focused on reducing stigma among providers through share workshops with favorable results [9, 10], while one focused on creating a “culture of support,” including supportive messages on women’s reproductive decisions, addressing stigma, and abortion-related information [11]. Women reported that the intervention improved their ability to cope with negative judgement by others. None of these articles assessed changes in women’s perceived stigma, quantitatively measured women’s experiences of abortion, or differences across health professionals and peer counselors. A Cambodian study found that women who engaged with counselors via mobile-phone post-abortion were significantly more likely to use contraception post-abortion versus women who did not speak with counselors [12]. Further examining the role of nurses and peer counselors and developing strategies to change women’s perceived stigma and experiences of care are critical to improve post-abortion outcomes.

Finally, person-centered care (PCC)—“care that is respectful of and responsive to, women’s values, preferences and needs”—is needed for abortion [13]. Limited PCC studies demonstrate promising results. In Nepal, an intervention on interpersonal communication promoted dialogue in communities enabling women to enhance communication within their households, specifically with partners [14]. In South Africa, a randomized controlled trial (RCT) on mobile phone SMSes to support women post-abortion reduced anxiety and stress post-abortion [15]. Intervention acceptability was also high as nearly all women in the intervention group recommended these SMSes to friends [15]. Other studies also demonstrate high acceptability of phone follow-up support among women who had early medication abortions (MA) [16]. While these interventions touched on aspects of PCC, none have explicitly focused on a comprehensive PCC approach for abortion.

Our study fills a critical gap in the literature by assessing the impact of a person-centered abortion care (PCAC) mobile phone-based intervention through peer counselors and nurses to improve social support, decrease abortion stigma, and decrease depression for women who had abortion-related procedures in private facilities. Specifically, this study assesses whether additional follow-up support post-abortion was beneficial to women’s sense of social support, abortion-related stigma, mental health, and whether there was a difference between the standard of care (MSK call center with no PCAC training) and the type of supporter (peer counselor versus nurse with PCAC training). The intervention includes two provider types: peer counselor and nurse to explore women’s preferred source of support. The peer counselor trained in PCAC normalizes the woman’s abortion experience and therefore reduce stigma whereas the nurse trained in PCAC provides the woman with trusted medical expertise. A study evaluating peer-led and adult-led sex education programs suggests that both approaches can play important yet different roles in improving sex education [17] and worth exploring the impact of peer education and health promotion further [18].

## Methods

### Intervention description

The University of California, San Francisco (UCSF), Marie Stopes Kenya (MSK), and Innovations for Poverty Action (IPA) conducted an RCT of a PCAC support intervention in Nairobi, Kenya in 2018.

This intervention was designed using a participatory approach based on qualitative data from focus group discussions and in-depth interviews of women who had an abortion-related procedure at an MSK clinic between April-October 2017. This pilot study received separate ethics approval from the University of California, San Francisco (UCSF) (IRB #15–18008). Thematic analyses of qualitative transcripts identified gaps in care, determined how different women wanted to be supported, and who they would want to provide support and when. One peer counselor (a woman who had an abortion previously) and one nurse then participated in the same three-day training on topics such as PCAC, abortion types and potential complications, phone/SMS etiquette, and privacy/confidentiality. Each interaction with participants utilized principals of PCAC including dignity/respect, autonomy, communication, trust, privacy/confidentiality, stigma/discrimination, and social support as previously described [13]. Follow-up calls and SMSes were designed to make sure there are no complications (and refer if so) and to see how women are emotionally healing using a semi-structured guide. While the aim of the follow-ups across arms was the same, the training emphasized how to vary the delivery based on role. For example, while asking the woman about her physical and emotional recovery, the peer counselor was trained to use her own experience to normalize the experience of the woman, while the nurse was trained to use a medical lens in her interactions.

The control group received the standard of care (SOC) from MSK: a phone call from MSK’s contact center within two weeks post-procedure and not trained in PCAC. The peer counselor and nurse arm received SOC plus additional support from a PCAC trained peer or nurse with three phone calls (24-hours, 1-week, and 3-weeks post-abortion), two SMSes (48 hours and 72 hours post-abortion), and support via WhatsApp, if initiated by the woman. All participants were informed of the different arms at enrollment and follow-ups were initiated by sharing whether they were speaking with a peer counselor or nurse.

### Study design and data collection procedures

Six female enumerators underwent a one-week training on the survey tool and conducted one-week piloting at MSK clinics. From August 27, 2018 through December 21, 2018, women were introduced to the study in collaboration with MSK providers if they: 1) underwent a surgical abortion (SA) or took the first MA pill at the clinic on the day of recruitment, 2) were at least 15 years old, 3) spoke English or Swahili, 4) owned a mobile phone with WhatsApp capability.

We assumed a sample size of 330 participants per arm as calculated in Stata 15MP. For more information on original sample size calculations, refer to S1 Appendix. It should be noted that due to an unexpected government ban on abortion services in Kenya during the time of the study, only 371 women were recruited into the study before the ban came into effect. We subsequently ran a power calculation with repeated measures to detect a difference across mental health scores, assuming a sample of 122 and 127 in each arm, two follow up measures, and standard deviations of 22.44 and 18.36. Given these assumptions, the power to detect a difference was 0.81.

At the time of recruitment, we conducted a baseline survey at the clinic in a private space on PCAC experiences, post-abortion family planning (PAFP) care, mental health, social support, and stigma. Randomization into one of three study arms occurred after completion of the baseline survey and was electronically generated by a randomization feature on the electronic data collection platform, SurveyCTO. After completing the baseline survey, women were notified by the enumerator on which study arm they were placed into and received airtime equivalent to approximately $1.50. A phone follow-up survey (2-weeks and 4-weeks) post-abortion was administered to assess study outcomes. Per protocol, three attempts were made before deeming participants unreachable for follow-up. After follow-up survey completion, women received airtime equivalent to approximately $0.50. The full study protocol is available in S1 Appendix.

### Measures

#### Outcomes of interest

Mental health was assessed at baseline and 2-weeks follow-up using the validated 5-item Mental Health Inventory (MHI-5) scale (Cronbach’s α = 0.82) [19]. The MHI-5 includes questions on how often women felt stressed, calm, sad, happy, and low in past weeks. Response options were adapted from a 1–6 range option to 1–4: 1 (all of the time) to 4 (none of the time) and subsequently added to obtain a total score ranging from 5–20 with higher scores indicating better mental health. Social support was measured at baseline and 4-weeks follow-up using the 18-item MOS Modified Social Support Scale (MSSS), which asks participants to report how often someone is available to provide various types of support from four domains: emotional/informational, tangible, affectionate, and positive social interaction (all domains have Cronbach’s α>0.91) [20]. We excluded three items related to positive interaction items that were irrelevant to our sample, resulting in a total of 15 items for social support. Response options for each item ranged from 1 (none of the time) to 5 (all of the time) and were totaled to obtain a total score from 18–90 where higher scores indicate greater social support. Finally, abortion-related stigma was assessed at baseline and 4-weeks follow-up using 3 items from the “worries about judgment” domain of the Individual Level Abortion Stigma (ILAS) scale [21], which ask women how worried they felt about others finding out about their abortion, people gossiping about them, and disappointing someone they love (Cronbach’s alpha ranged from 0.73 at baseline and 0.76 at 4-weeks follow-up). Response options for each item ranged from 1 (not worried) to 4 (extremely worried) so that total scores ranged from 0–9 whereby higher scores indicate greater worry about stigma. To avoid interviewee fatigue, we structured our 2- and 4-weeks follow-up surveys to take 15–20 minutes and administered the MHI at 2-weeks and the MSSS and abortion stigma scale at 4-weeks.

#### Sociodemographic variables

Demographic characteristics, such as age, relationship status, religion, employment status, educational attainment, and pregnancy history, were collected at baseline. Information on the type of abortion procedure received was also collected. The two abortion procedures are very distinct. MA involves the use of a drug or a combination of drugs to terminate a pregnancy (i.e. mifepristone or mifepristone followed by misoprostol) and is typically completed over a one or two day period. MVA, on the other hand, is typically completed at the health facility in a single appointment. Because women’s satisfaction may be associated with their preferences and experiences of taking the pill at the health facility or at home [22], we categorized the type of abortion procedure as one of the following: 1) MA, 1^st^ pill (self-administered at MSK clinic); 2) MA, 2^nd^ pill (self-administered at MSK clinic); 3) MA 1 pill (self-administered at home); or 4) surgical abortion/medical vacuum aspiration (MVA).

#### Analyses

Participant characteristics at baseline were summarized by study arm using simple frequencies (age and pregnancy history variables recoded to be categorical) with corresponding p-values derived from Pearson’s chi-square tests. Mental health, stigma, and social support scores at baseline and follow-up were rescaled to be out of 100. T-tests were used to calculate differences in means for mental health, stigma, and social capital scores at baseline and follow-up and to examine pre-post changes in scores for each study arm. We also examined these differences using a non-parametric Wilcoxon signed-rank test and all conclusions were the same as the t-tests. Assumptions for t-tests were checked and no violations were observed. For simplicity of interpretation, we present t-test results. We used the Kruskal-Wallis one-way ANOVA test to assess the effect of each intervention on changes in mental health, stigma, and social support outcomes from baseline to follow-up. We compared each study arm to the control arm. Because there were no statistically significant differences between the groups at baseline, we did not control for socioeconomic characteristics or other covariates. All analyses were conducted using StataSE, version 15.1.

### Ethical approvals

The Committee for Human Research of UCSF, the Kenya Medical Research Institute and Marie Stopes International’s Ethics Review Board approved this RCT. All interested and eligible participants provided written informed consent prior to any study procedures. Women who were older than 15 and younger than 18 were also consented without parental approval if they were considered a “mature minor.” Mature minors are those who are married, a mother, pregnant, or a head of household. Because women had consented to a safe abortion procedure, we assume that they are a mature minor. While mature minors were eligible for our study, there were no participants younger than 18 in the study.

The study was retrospectively registered at ClinicalTrials.gov (NCT#: NCT04206514). UCSF follows FDAAA registration requirements, which requires clinical registration of studies of FDA regulated drugs, biological products, and medical devices. This study did not meet the requirement of needing registration (i.e. did not contain a drug or biological product), and therefore, was not registered until requested by the publication.

## Results

**Fig 1:**
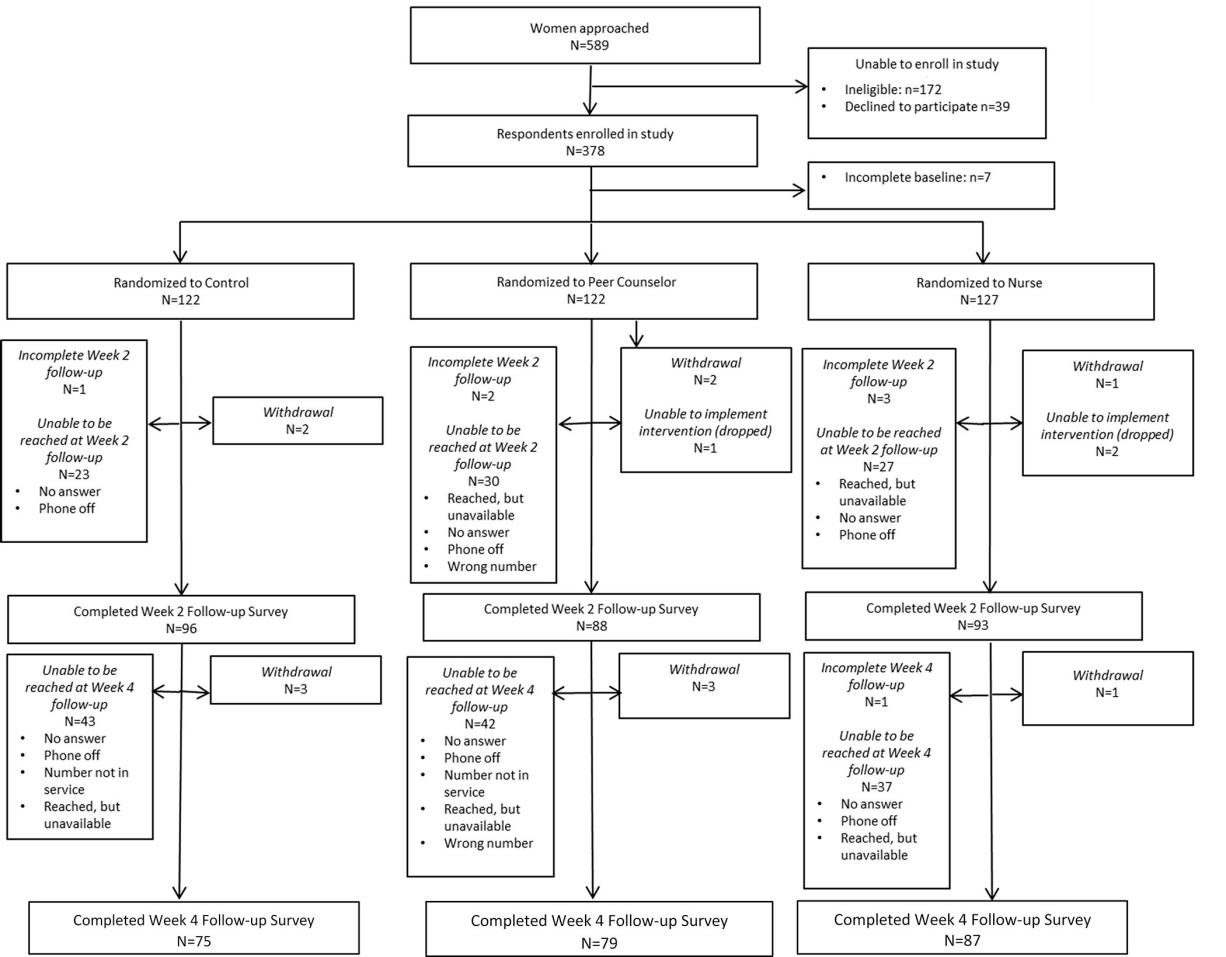
Flowchart of participants (CONSORT diagram).

Of the 378 women enrolled, 371 completed baseline and were randomized into one of three study arms: control arm (n = 122), peer counselor arm (n = 122), and nurse arm (n = 127). The overall retention rate was 74.7% and 65.0% at the 2- and 4-week follow-up, respectively. Attrition did not significantly differ by study arm and was predominantly related to not being able to reach participants (e.g. phone off, no answer, reached but unavailable, wrong number or number not in service), participants who voluntarily withdrew from the study, and participants who were unable to be reached by MSK to implement the intervention (dropped from study) (Fig 1).

Table 1 presents participant characteristics; no statistically significant differences were detected across study arms. Summary statistics and bivariate analyses from baseline to follow-up across the three study arms are provided in Table 2. Mental health scores were significantly higher across all study arms (improved mental health) at two-week follow-up compared to baseline. Stigma scores decreased by an average of seven points in intervention arms at follow-up versus baseline. Similarly, social support scores among the intervention arms significantly increased from baseline to four-week follow-up. No significant differences in stigma and social support scores were detected in the control arm.

**Table 1:** Participant characteristics stratified by study arm.

*Characteristic*	Control Arm (n = 122)	Peer Counselor Arm (n = 122)	Nurse Arm (n = 127)	Total (N = 371)
	n (%)			
**Age, years**				
Less than 20	13 (10.7)	11 (9)	8 (6.3)	32 (8.6)
20–24	48 (39.3)	41 (33.6)	55 (43.3)	144 (38.8)
25–29	39 (32)	32 (26.2)	29 (22.8)	100 (27.0)
30–34	11 (9)	18 (14.8)	23 (18.1)	52 (14.0)
35 or older	11 (9)	20 (16.4)	12 (9.4)	43 (11.6)
**Married, partnered, or cohabitating**				
Yes	28 (23.0)	27 (22.1)	29 (22.8)	84 (22.6)
No	94 (77.0)	95 (77.9)	98 (77.2)	287 (77.4)
**Religion**				
Christian	118 (96.7)	118 (96.7)	117 (92.1)	353 (95.1)
Muslim	2 (1.6)	3 (2.5)	7 (5.5)	12 (3.2)
None	2 (1.6)	1 (0.8)	3 (2.4)	6 (1.6)
**Currently employed for pay**				
Yes	73 (59.8)	79 (64.8)	67 (52.8)	219 (59.0)
No	49 (40.2)	43 (35.2)	60 (47.2)	152 (41.0)
**Education**				
Primary or less	14 (11.5)	13 (10.7)	10 (7.9)	37 (10)
Secondary or vocational	43 (35.2)	47 (38.5)	57 (44.9)	147 (39.6)
College or University	65 (53.3)	62 (50.8)	60 (47.2)	187 (50.4)
**Number of pregnancies**				
1	58 (47.5)	56 (45.9)	62 (48.8)	176 (47.4)
2	34 (27.9)	29 (23.8)	28 (22.0)	91 (24.5)
3	17 (13.9)	18 (14.8)	15 (11.8)	50 (13.5)
4	8 (6.6)	9 (7.4)	12 (9.5)	29 (7.8)
5 or more	5 (4.1)	10 (8.2)	10 (7.9)	25 (6.7)
**Number of live births**				
0	69 (56.6)	65 (53.3)	72 (56.7)	206 (55.5)
1	32 (26.2)	25 (20.5)	24 (18.9)	81 (21.8)
2	15 (12.3)	16 (13.1)	18 (14.2)	49 (31.2)
3 or more births	6 (4.9)	16 (13.1)	13 (10.2)	35 (9.4)
**Number of children**				
0	71 (58.2)	64 (52.5)	74 (58.3)	209 (56.3)
1	30 (24.6)	28 (23.0)	24 (18.9)	82 (22.1)
2	15 (12.3)	14 (11.5)	16 (12.6)	45 (12.1)
3 or more children	6 (4.9)	16 (13.1)	13 (10.2)	35 (9.4)
**Type of abortion received**				
Medical Abortion, 1st pill (self-administered at MSK center)	55 (45.1)	52 (42.6)	56 (44.1)	163 (43.9)
Surgical Abortion/Medical Vacuum Aspiration	52 (42.6)	62 (50.8)	52 (40.9)	166 (44.7)
Medical Abortion, 2nd pill (self-administered at MSK center)	9 (7.4)	3 (2.5)	10 (7.9)	22 (5.9)
Medical Abortion, 1 pill (self-administered at home)	6 (4.9)	5 (4.1)	9 (7.1)	20 (5.4)

**Table 2:** Mean (standard deviation) mental health, stigma, and social support scores at baseline (BL) and follow-up (FU).

*Primary Outcome*	Control Arm			Peer Counselor Arm			Nurse Arm		
	BL Mean (SD)	FU Mean (SD)	Mean Diff (FU-BL) (SD)	BL Mean (SD)	FU Mean (SD)	Mean Diff (FU-BL) (SD)	BL Mean (SD)	FU Mean (SD)	Mean Diff (FU-BL) (SD)
**Mental Health^**	62.24 (17.83)	77.81 (15.72)	15.57 (22.44)***	61.70 (15.01)	76.36 (14.79)	14.66 (18.36)***	68.23 (15.49)	79.41 (14.12)	11.18 (17.06)***
**Stigma~**	28.27 (25.91)	25.87 (23.49)	-2.40 (23.59)	33.04 (29.93)	25.95 (25.75)	-7.09 (20.20)**	32.41 (26.85)	25.40 (24.25)	-7.01 (28.70)*
**Social Support~**	69.30 (21.68)	72.30 (20.05)	3.00 (16.46)	70.48 (18.56)	76.86 (18.43)	6.38 (19.09)**	70.59 (19.55)	75.92 (18.96)	5.33 (15.59)**

^Follow-up at Week2; N = 96 for Control Arm; N = 88 for Peer Counselor Arm; and N = 93 for Nurse Arm

~Follow-up at Week4; N = 75 for Control Arm; N = 79 for Peer Counselor Arm; and N = 87 for Nurse Arm

*p < .05

**p < .01

***p < .001

A Krukal-Wallis test compares the effects of each study arm with the control arm (Table 3). The nurse arm improved mental health scores from baseline to week two; however this was only marginally significant (p = 0.059). The nurse arm also lowered stigma scores from baseline to week four, and this was marginally significant (p = 0.099). The peer counselor arm did not have statistically significant effects.

**Table 3:** Kruskal- Wallis one-way ANOVA test to compare effects of each study arm.

Outcome Variable	Control vs. Peer Counselor Arm	Control vs. Nurse Arm
	Chi2 (p-value)	Chi2 (p-value)
Mental Health	0.223 (0.637)	3.567 (0.059)
Stigma	2.420 (0.120)	0.121 (0.099)
Social Support	0.200 (0.157)	1.845 (0.174)

## Discussion

To our knowledge, this is the first study to develop a PCAC, mobile phone-based intervention for post-abortion clients in Kenya. We found that women who received support from a nurse reported marginally improved mental health and decreased perceived stigma. While our study found women reported increases in perceived social support among the intervention arms compared to the control arm, this was not statistically significant.

The peer counselor arm was included to normalize women’s abortion experience (i.e. reduce stigma), but our results suggest that the nurse arm marginally statistically significantly improved mental health and decreased stigma compared to the control (p<0.1). Potential explanations for nurses being more effective in reducing stigma and improving mental health include health-related misconceptions of abortions including abortions increasing the risk of breast cancer and leading to infertility [23]. A medically trained nurse may be better positioned to clarify these issues and thus decrease perceived stigma, and in turn, improve mental health [21]. Additionally, small-scale studies in Kenya found that women expect to be stigmatized by their own healthcare providers [24, 25]; therefore, interactions with PCAC-trained nurses may ease anxiety related to disrespect. The ability of nurses to give health-related information and be trained in PCAC provides a unique role for this cadre of staff. During the qualitative intervention development, women expressed the need for informational and social support, which the nurse and peer counselor were intended to provide. Other studies on maternity care and emergency situations found that training on respectful care integrated with clinical training improved women’s experiences of care [26]. Future studies may combine clinical and PCAC training to tackle abortion-related misconceptions, provide critical information to women, and support their post-abortion care experiences.

We found statistically significant improvements in mean levels of mental health when assessing changes in mental health across time within each of the three study arms individually. Women were surveyed directly post-abortion in the health clinic; therefore, they may experience immediate relief after the procedure aligning with existing literature suggesting that women consistently report improved mental health post-abortion [27–29]. Other studies found decreased depression and increased self-esteem among women pre- and post-abortion [29]. Additionally, there was no effect of the intervention on social support, but social support was statistically significantly improved from baseline to follow-up for both intervention arms.

There are several study limitations. First, the intervention included only one peer counselor and one nurse. While they received the same PCAC training and had guides for follow-up support, we cannot definitively conclude that the only difference in their interactions with women stemmed from their professional backgrounds. A study with a larger intervention is needed to validate these results. Second, the study was conducted in private clinics and women’s experiences may not be generalizable to women seeking abortion-related services elsewhere. Third, the study faced interruptions in Kenya’s legally restrictive context, including a temporary national suspension of safe abortion provision across all service delivery points, which resulted in the study ending early [30]. However, power calculations confirmed that study sample size was sufficient for analyses to detect differences across study arms. Future studies with larger sample sizes are needed to compare results across abortion type (i.e. MA vs. surgical abortions, etc.) as other studies found that PCAC may be higher for surgical abortions clients compared to MAs [31]. Other intervention challenges included high loss to follow-up due to inability to reaching respondents by phone. Future studies should account for this loss to follow-up. Moreover, due to topic sensitivity, 7% of women refused to participate in the study. Therefore, those who consented may be different than those who refused. Future studies should ask women why they refused to participate in abortion studies to determine how refusals may influence results. Similarly, while loss to follow-up was similar across arms, women who were unreachable at follow-up may be different from women who completed the intervention.

### Implications of the study

Future studies should use a theory-driven approach, including concepts of PCAC for reproductive health [13], and conduct formative interviews with women themselves to guide intervention development and implementation. The results suggest that incorporating PCAC training/counselling and adapting existing clinical infrastructure may improve women’s experiences post-abortion, improve mental health, and reduce perceived stigma. This may be feasible even in resource-limited settings given that nurses are an integral part of the staff structure. Our results suggest an additional training on PCAC is warranted, perhaps incorporated into existing provider trainings on sexual and reproductive health. Mobile phone-based interventions are popular given the ability to make calls and SMSes at low cost, while providing continuous respectful and supportive follow-up support post-abortion. In particular, mobile-based nurse support may be a promising approach to reduce stigma and support women post-abortions.

## Supporting information

S1 Checklist (DOC)

S1 Appendix Full study protocol. (DOCX)
