# Supplementary material for: Improving stigma and psychosocial outcomes among post-abortion Kenyan women attending private clinics: A randomized controlled trial of a person-centered mobile phone-based intervention
Source: PLoS One. 2022 Jun 24;17(6):e0270637. doi: 10.1371/journal.pone.0270637 (PMC9232159; doi:10.1371/journal.pone.0270637)
Supplement: S1 Appendix — (DOCX) [file pone.0270637.s002.docx]

1. **TITLE OF THE PROJECT**: Strengthening Patient-Centered Accessibility, Respect, and Quality of Care for Post Abortion Care through Peer and Nurse Based Phone Support (SPARQ Project)

**Protocol Version 9:** **7 August 2018**

1. **INVESTIGATORS AND INSTITUTIONAL AFFILIATIONS:**

University of California, San Francisco (UCSF)

Principal Investigator: May Sudhinaraset, PhD, UCSF

Marie Stopes International (MSI) and Marie Stopes Kenya (MSK)

Co-Investigator, Edward Kubai from MSK

Co-Investigator, Olivia Nuccio from Marie Stopes International

**Other Study Personnel (Non-Investigators) with Access to PII:**

University of California, San Francisco (UCSF)

Beth Phillips, Research Analyst

Avery Seefeld, Program Coordinator

Innovations for Poverty Action (IPA) Kenya

Ginger Golub, Research Manager

James Opot, Research Associate

1. **ABSTRACT:**

Unsafe abortion remains a problem globally, accounting for approximately 13% of all maternal deaths worldwide. Africa is disproportionately affected, accounting for nearly two-thirds of all abortion-related deaths (World Health Organization, 2011). Unsafe abortions have been estimated to account for between approximately 35% to as high as 78% of maternal mortality in Kenya (Desai et al., 2013; Oyieke, Obore, & Kigondu, 2006; Ziraba, Madise, Mills, Kyobutungi, & Ezeh, 2009). In Kenya, abortion is allowed if there is need for emergency treatment, or the life or health of the mother is in danger. The Constitution states that every person has the right to the highest attainable standard of health, including reproductive health care (Republic of Kenya, 2010). Despite the law, barriers exist to provision of and access to safe abortion services. Community support groups for abortion clients and support from health professionals could work to address stigma and feelings of alienation and isolation among women post abortion. Many patient-centered, evidence-based interventions used for other health issues are transferrable to social support for abortion (Upadhyay, Cockrill, & Freedman, 2010). Past peer-led support interventions have suggested that identifying trusted and appropriate leaders is critical in the success of interventions. However, women in Kenya receive information about abortion services from a number of disparate sources. For example, Maries Stopes Kenya (MSK) has observed that women first receive information about abortion services from informal networks, including from the Internet (i.e. Google.com, etc.) and traditional birth attendants. Preliminary research data from UCSF/MSK suggests that safe abortion and post abortion care (SA/PAC) clients lack information about family planning and pain related to abortion (UCSF, IPA, & MSK, n.d.), and that (SA/PAC) clients received less support than they desired. At a community level, social stigma around abortion services is high. Stigma about abortion remains high in communities and this creates a significant barrier to provision of and access to abortion services. Relatedly, there are varying degrees of support for SA/PAC clients. Research is needed to test the feasibility and acceptability of a community support group for (SA/PAC) clients in Kenya. Towards this goal, we will implement a randomized control trial, mixed-methods approach to design and pilot test a community-based intervention. This project aims to test a person-centered care (PCC) follow-up component with women who have recently received an SA/PAC. MSK will utilize existing/hire nurses and peer leaders to follow-up with women who have recently had an SA/PAC at their clinic. These nurses and peer leaders will be trained in PCC and will both follow-up with women to make sure there are no complications and to see how they are emotionally healing. The project findings will lead to large-scale implementation and roll out of this intervention in MSK clinics.

1. **INTRODUCTION/BACKGROUND:**

Kenya has an extremely high abortion mortality rate of 266 deaths/100,000 live births (Brookman-Amissah, 2004). While unsafe abortion contributes 13% to global maternal mortality, and 18% to East African maternal mortality, this number skyrockets to more than 33% in Kenya, nearly triple the global average (Magadi MA, 2003; Evens E, 2013). Further evidence of unsafe abortion in Kenya is provided by Mohamed et al. in a 2015 mixed methods study. The researchers estimated the incidence of induced abortion in Kenya in 2012 to be about 464,000, which translates to a rate of 48 induced abortions/1,000 women aged 15-49 years old (Mohamed SF, 2015). Important risk factors for unsafe abortion (and unintended pregnancy) are: young maternal age, ethnicity, and level of educational attainment, employment status, type of housing, and most significantly marital status (Magadi MA, 2003; Ikamari L, 2013). In the Kenyan context, traditional sociocultural norms and religious beliefs continue to stigmatize and condemn abortion, such that even if it were legally accessible (which is interpreted differently given uncertainties in a recent law), access would be limited (Magadi MA, 2003). The ramifications of these effects are that abortion remains widespread and it is most often performed clandestinely and unsafely (Brookman-Amissah E, 2004).

1. **JUSTIFICATION FOR THE STUDY:**

Post-abortion care services and support are critical in the survival of women from unsafe abortions: Past work suggests that women experience low levels of social support, particularly during the post-abortion period, due to perceived community stigma and lack of information from trusted support networks. While past research suggests that abortions can be a positive experience for the majority of women, some women may experience stress during post-abortion period resulting in feelings of alienation and isolation (Charles, Polis, Sridhara, & Blum, 2008). Recent evidence shows addressing stigma among women seeking abortions may notably lower their psychological distress (Steinberg, Tschann, Furgerson, & Harper, 2016). Moreover, the post-abortion period may significantly improve post-abortion family planning uptake and thus provide an opportunity to reduce future unintended pregnancies (Ceylan, Ertem, Saka, & Akdeniz, 2009). If women internalize abortion stigma post abortion this can perpetuate the problem of unsafe abortion at a community level. It is therefore critical to provide social support interventions for women who have undergone abortion services. Additionally, abortion stigma plays a large role in women's decision on whether to have a safe or unsafe abortion (Yegon, Kabanya, Echoka, & Osur, 2016).

Social network-based interventions have already shown promise, including improving sexual behaviors (Bull, Levine, Black, Schmiege, & Santelli, 2012), cancer support (McLaughlin et al., 2012), HIV (Amirkhanian, Kelly, Kabakchieva, McAuliffe, & Vassileva, 2003; Kelly et al., 1997; Latkin, Sherman, & Knowlton, 2003; Pulerwitz, Michaelis, Verma, & Weiss, 2010), and drug use (Cuijpers, 2002). Peer and adult-led sex education in schools (Mellanby, Newcombe, Rees, & Tripp, 2001) and on online social networking sites (SNSs) (Bull et al., 2012; Levine, 2011) has led to improvements in sexual behaviors. Technology-based platforms, in particular, have shown to be potentially effective for reducing adolescent sexual risk behaviors (Allison et al., 2012; Lightfoot, Comulada, & Stover, 2007). This is particularly relevant for transnational communities that heavily rely on technology communication platforms to maintain social ties. Health programs on SNSs increase social capital and social support among young people (McLaughlin et al., 2012) and increase sexual health knowledge (Bull et al., 2012). Peer education programs are cost-effective and empower hard-to-reach populations (Turner & Shepherd, 1999).

In 2017, we conducted qualitative formative research using a participatory approach with women who received SA services at MSK to develop and inform the proposed intervention. Qualitative interviews identified what women preferred and needed in terms of the type of platform (I.e. In-person, phone, internet, etc.), type of support (I.e. Information, emotional, instrumental), source of support (I.e. Health professional, peer, etc.), and duration of intervention for their post-abortion care.  From focus group discussions and in-depth interviews, we found that one of the most challenging periods for women was during the post-abortion period, including lack of social support and feelings of stigma and guilt. Additionally, women suggested that while in-person support would be helpful, the most feasible and convenient platform to use would be phone-based support either with a woman who has been through the same experience (a peer counselor) and with a health professional to provide needed information and social support. These findings led to the current proposed social support intervention using What’s App text messaging and training peer counselors and health professionals on person-centered care.

For this study, we define Safe Abortion as the procedure used to terminate a pregnancy within the confines of the Kenya Law. All safe abortions are provided within the legal framework of Kenya Abortion Law. Within the legal framework means that a safe abortion is provided to a woman when in the opinion of a trained health professional, there is need for emergency treatment,or the life or health of the mother is in danger.

1. **OBJECTIVES**
   1. **GENERAL OBJECTIVE:** The main objective is to improve the quality of care received by women post-abortion in Kenya.
   2. **SPECIFIC OBJECTIVE:** To evaluate an support intervention aimed at providing clients who have received an SA/PAC procedure with person-centered follow-up care.
2. **DESIGN AND METHODOLOGY:**
   1. **Study Site (Geographical):** SA/PAC clients will be recruited post procedure from MSK clinics in Nairobi County. These clients will be followed-up via phone calls and text messages.
   2. **Study Populations**
      - **Criteria for inclusion of subjects:** women of reproductive age who had an SA/PAC service that day at an MSK clinic will be included in the study. Women must be willing and able to comprehend and give informed consent (verbally read consent to respondents and respondents will provide written consent), and able to communicate in English and/or Swahili. Women must have a personal cell phone with WhatsApp capabilities, have access to this phone for the next four weeks, and be willing to be communicated with MSK and IPA after their SA/PAC service via SMS**/**WhatsApp and phone calls.

Those under 18 years of age are considered a mature minor and therefore are able to participate. Mature minors are considered capable of consenting to the SA/PAC procedure and therefore we believe they should also be considered capable of consenting to participate in this study.

Nearly all MSK clients within Nairobi can speak in English and/or Swahili therefore we do not anticipate that this criteria will exclude women from participating in the research.

- - - **Criteria for exclusion of subjects:** Women who are not receiving an SA/PAC service from an MSK clinic will be excluded from the study. Women who are not of reproductive age will be excluded from the study. Women who do not have a personal cell phone that is compatible with WhatsApp, will not have access to this phone for the next four weeks, or do not wish to be contacted by MSK and IPA will be excluded from this study. Women who cannot or do not provide informed consent to participate will be excluded from the study. Women who are unable to communicate in English and/or Swahili will be excluded from this study. Women who have already participated in this study will not be eligible to participate again.
    - **Rationale for animal use and justification for animal species chosen:** Not applicable
  1. **Sampling**
     - **Sample Size Determination:** we will enroll up to 1,000 clients in Nairobi who have just had SA/PAC service at a MSK clinic. Clients will be notified of this project and those who agree to participate will be randomly placed in one of three arms, with each arm of this study having up to 330 participants. We ran these sample size calculations using Stata 15MP. Using the *sampsi* command, we used parameters from existing literature on person-centered care. This sample is based on existing person-centered care mean of 59.8 for delivery patients (see sample size rationale below). A sample size of 330/study arm would allow us to detect a five point difference between the intervention and control group with a power of at least 0.90 and standard deviation of 13, accounting for repeated measures and 50% attrition from baseline to endline. This is feasible within an approximate five month period, given the high volume of clients in MSK clinics. For example, across MSK clinics within Nairobi County, data from last year show that the average number of SA/PAC clients is approximately 617 per month. Therefore, taking into account those who are not interested and clients at the clinic when researchers are not present, we should be able to reach our target of 330 respondents per arm over the course of approximately five months.
     - **Sample size rationale:** We assume a sample size of 330 participants per arm. We conducted a power analysis of detecting a five point difference in average person-centered care score based on existing scales among maternity delivery clients in Kenya. No data exists for person-centered care among abortion clients. We used Stata 15MP using the sampsi command. The parameters used for the sample size calculation was based off of existing literature. We assume standard devision of 13 and 20 for pre/post responses, respectively, account for repeated measures and attrition of 50%. We used 50% attrition based on IPA’s extensive experience conducting follow-up surveys and MSK’s experience working with SA/PAC clients.

**Sampling Procedure:** Clients will be recruited from up to six MSK facilities located in Nairobi County: KenCom, Kenyatta Market, Eastleigh, Kibera, Kangemi, Pangani. Women who come in for an SA/PAC will be notified about this program by MSK clinic staff and asked if they would like to participate. If so, IPA field staff will provide additional details of this program. If the client is eligible and interested, the client will be asked to provide consent and then she will be randomly assigned on a rolling basis to one of the three study arms. Random assignment to one of the three study arms will be electronically generated by a randomization feature on the electronic data collection platform (i.e. SurveyCTO). Since baseline occurs on a rolling basis, respondents will be followed up while others are newly enrolled. Therefore, we will continue sampling until at least 200 respondents per study arm have completed the final follow-up survey (ie. 1 month post-procedure).

All women who agree to participate and are assigned into different arms of the study will also be asked to provide baseline data following their SA/PAC procedure and provide their contact information to participate in follow-up surveys that will be conducted over the phone.

**e. Procedures**

**Methodology: To test an SA/PAC support intervention aimed at providing patients with person-centered follow-up care**

| Summary of Methods: Upon receiving an SA/PAC service at an MSK clinic, clients will be invited to participate in a post-abortion support intervention. This intervention will have three arms: a control group that receives standard follow-up care, a group that receives follow-up care by a nurse trained in PCC, and a group that receives follow-up care from a peer who has also had an SA/PAC and been trained in PCC. We will then evaluate the post-abortion experience of the participants in each of these three arms. |
| --- |

IPA, in collaboration with UCSF, will train female research assistants on quantitative surveying and public health ethics and confidenitality. IPA research assistants will be at MSK facilities in Nairobi. When a woman visits a MSK clinic seeking SA/PAC service, her provider or other MSK staff member will introduce this study and ask the woman if she is interested in speaking with the IPA research assistant about participating. If the woman is interested in the study, the MSK staff member will refer her to the IPA research assistant (RA) after her procedure. This RA will read from a recruitment script (See Appendix A) that will provide information on the study, and ask a short set of questions to confirm if a woman is eligible. Any discussion about the study (at initial introduction by the provider and at study recruitment and consenting by the IPA RA) will be done in a private setting (in an unused room within the facility grounds). RAs will be trained on the importance of finding a setting where privacy is maintained during the introduction, recruitment, consenting process, and data collection.

**Eligbility criteria**

- Women of reproductive age
- Had an SA/PAC procedure at an MSK clinic that day
- Has her own cellphone that is WhatsApp compatible and will have access to this phone for the next four weeks
- Willing to receive SMS messages and phone calls from an MSK**/**IPA professional
- Able to communicate in English and/or Swahili
- Willing and able to comprehend and give informed consent
- Willing to participate in a baseline survey immediately after providing consent
- Willing to be followed up by phone at two weeks and four weeks post procedure for a 15-minute survey at each time-point
- Has not already participated in this study

For each participant:

1. **Recruitment:** During the pre-service consultation at a MSK clinic, the MSK provider will mention that they are conducting an intervention for post SA/PAC clients. The provider will explain that clients are free to participate but not obligated; she will still receive the same standard care if she participates or not. If the client is interested in participating the provider will connect the client with the IPA research assistant after the procedure. In a private space, the trained IPA research assistant will then explain the objectives of the study and eligibility criteria for study participation (as outlined above) to the potential participant. The RA will explain that the study participation will not affect the services the woman receives at MSK and she can drop out at any point. Additionally if she does not want to participate in the study at all she will still receive the same care she would normally get as a client of MSK. If the participant is eligible and willing to participate, the IPA research assistant will seek informed consent. The research assistant will be using a recruitment script that clearly outlines the elibility and consenting process.
2. **Consent**: In a private space, the recruited participants will be taken through the informed consent process to ensure the woman understands the purposes and procedures of the study, that her confidentiality will be kept, as well as the study being voluntary and she is free to withdraw with no consequences at any time. Participants can withdraw their data from the study at any time, including after the surveys have been completed. If the participant agrees, she will provide written consent. If the participant does not wish to participate, she will be free to continue with her remaining care services and/or to leave the facility with no consequences.
3. **Contact Information:** Following informed consent the research assistant will electronically record contact information for the participant so that IPA can reach her for follow-up surveys. This information will be encrypted, password protected, and stored on a secure server with limited access to ensure confidentiality.
4. **Baseline Survey –** Once a woman consents, the IPA research assistant will conduct a baseline survey which should take approximately 40 minutes. This baseline survey will be conducted in the same private room where consent took place.

Following the baseline survey, the IPA research assistant will randomize the women to one of the three arms of the study. The IPA RA will then share with MSK’s call center the client’s patient ID number, which study arm she has been randomized, and the contact number the patient wants used during the study period so that they can provide the correct follow-up intervention. This information will be shared with MSK to confirm that the names, ID, and numbers all match, and update any information that was provided most recently by the client to IPA RA. Since MSK has the client’s information on a central server they will not need to transfer names unless there are discrepancies between the contact information provided by the patient.

1. **Post Intervention Surveys –** IPA research assistants will then follow-up with these women over the course of the intervention to conduct follow-up surveys at approximately 2 weeks and approximately 4 weeks post SA/PAC. These surveys will focus on the clients’ abortion experience and their post SA/PAC care. These post-surveys will last approximately 15 minutes and will occur over the phone. When IPA contacts these women for the follow-up surveys they will ask if she is free and able to speak openly. If she is not, they will schedule a time to call back that is more convenient for the client. If IPA does not reach a client they will not leave a voice message but send a genereic text message asking if there is a time that would be convenient to speak. IPA will follow-up with clients three time before deciding they are lost to follow-up. The text message is as follows: “We/IPA are trying to reach you in regards to a research study. Please call us when you are free if you are still interested in participating.”
2. **Incentives:** After completion of baseline, 150 Kenyan shillings of airtime (approximately equivalent to $1.50) will be given to respondents as reimbursement of transport. Respondents will receive 50 Kenyan shillings of airtime for each follow-up survey.

For respondents participating in this study: In the event that a respondent confesses to rape or incest, the study team will follow procedures laid out by MSK:

- The interviewer will refer the client to MSK’s service provider in the center where the interview is being held
- the service provider will be able to talk to the client and refer them to the Nairobi Women’s Hospital or a nearby alternative for counseling related to gender-based violence.
- In the event that a respondent expresses experiencing anything untoward by an MSK staff member, the study team will again follow MSK procedures for escalating complaints to MSK human resources, if the respondent wishes to do so.

*Intervention description:*

MSK in collaboration with UCSF will train nurses and peer leaders on person-centered care for SA/PAC clients. Women who go to an MSK clinic for a SA/PAC procedure and agree to participate in our study will be randomly assigned (on a rolling basis) to one of the three arms of this study.

1) **Control:** The first arm is the control group, where women will receive MSK’s standard follow-up care.

2) **PCC-trained Nurse:** The second group of women will receive a phone call at 24 hours after her procedure from a nurse trainined in person-centered care. She will then receive a SMS message through MSK’s existing messaging system 48 hours after her procedure. This message will be designed to let women know that MSK is available if they have any questions or concerns and that they are not alone. This message will also have a number that women can WhatsApp if they would like to continue the conversation over text. If women choose to reach out to MSK, WhatsApp is encrypted to ensure confidentiality of any information shared during these interactions. In addition, a nurse trained in PCC will follow up with these women and provide them with informational and emotional support (the nurse will call these women at 24 hours, 1 week after the procedure, and again at and three weeks after their SA/PAC).

The texts they will receive as part of the intervention are through MSK’s current SMS system, which is encrypted but does not allow two-way communication. Therefore, the peer counselor/nurse will provide a WhatsApp number women can use to continue communication that remains confidential since it is encrypted (regular SMS are not).

3) **PCC-trained peer:** The third group of women will receive the same phone calls and messages as the second group but instead of a nurse following up with them, a peer who has had an SA/PAC and been trained on PCC and abortion recovery will follow-up with these women (calling them at 24 hours, 1 week after the procedure, and again at and three weeks after their SA/PAC).

- These PCC-trained peers will be recruited by the MSK Call center and told about this intervention/evaluation and their potential role. During the recruitment process, “peer counselors” will be notifed that their participation is completely voluntary and that they can withdraw participation at anytime. They will also be made aware pior to accepting the position that in the role as peer-counselor they need to be comfortable discussing their own SA/PAC experience and that of the women they are speaking. Resulting in other people knowing that they had an abortion. However they will not be required to share their true identy with the women they are calling and they will be working from MSK and using MSK equipment so as much as possible their identify will be protected. These peer counselors will also be trained by MSK on the different components of this intervention and evaluation, SA/PAC, person- centered care, their role, and how to escalate situations they are uncomfortable handling so that they will be positioned to support women dealing with their decision but will not be in a situation to encourage a women to receive an abortion.
- Since MSK will hire peer counselors as MSK staff and pay them for their work we will not consent peer counselors.
- Peer counselors will be supervised and monitored by the Program Coordinator managing the rollout of this intervention. The Program Coordinator will be managed by Edward Ikiugu an MEI Officer at MSK and one of the PIs of this intervention. The peer counselors will work from the MSK facility in shifts and will be given MSK phones to use while at MSK (these phones will never leave the facility and the peer counselors will not contact women on their personal phones). Peer counselors will be asked to track their conversations with women and at the end of each week the peer counselors will meet as a group with the Program coordinator to discuss how their calls have gone, anything they had trouble addressing, and best practices they would like to share with the other peer counselors. Since all of the work the peer counselors are doing will be done at MSK the risk of participating as a peer counselor is minimized.

**Procedures for Data Verification & Validation**

Survey enumerators will be trained for approximately one week prior to the start of each round of data collection. The training will include comprehensive conceptual orientation of the topic of abortion, the study questionnaires and survey techniques. During data collection, field supervisors will aim to directly observe and/or backcheck approximately 15% of the interviews. Refresher training will be provided on an as-needed basis. Field supervisors will also ensure completeness of the data collected by the field staff before its transfer to a secure data server. The project data manager will perform extensive range and consistency checks, thus every effort will be made to develop a clean data set eliminating every possible error. Daily downloads of data collected will enable the monitoring team to promptly identify, check and address possible errors.

Each quantitative survey will be conducted by researchers who will be trained and guided by a senior researcher to ensure that surveys are conducted effectively.

1. **DATA MANAGEMENT AND ANALYSIS:**
   1. **Data management**
      - All data collected will be kept strictly confidential. No individuals will be identified in any publication.
      - Surveys will be collected electronically on tablets using SurveyCTO programming. The collected data will be uploaded to a secure server, survey data will never be stored on the tablet or smart phone. Any digital data will be encrypted, password protected, and stored on a secure server.
      - Only PIs and research assistants will have access to the databases resulting from surveys during the study. Research assistants hired for the project will be required to complete Human Subject Training (NIH or equivalent), and a Marie Stopes International Confidentiality Agreement. The databases will be password protected. The respondents will be informed of the confidential nature of the data collection.
   2. **Data Storage:** All field survey data will be backed up on a bimonthly basis and maintained on a secure server managed by IPA. During analysis, data will be stored in a secured server at UCSF for 5 years which will provide, long-term storage for research data. The data will be stored in password protected directories. The stored data at each server will be anonymized at the time of initial collection and storage, and no personal data will be included which could lead to disclosure of our study participant identities. All paper records, including consent forms, will be kept under lock and key at IPA offices in Kenya, with restricted access.

Data protection and confidentiality measures:

- Data on individuals will be stripped of their identifying information and subjects will be given a unique numeric code which will be used to identify them.
  - At conclusion of the survey and randomization before exiting the survey, the RA will be prompted to approve the participant ID, which will automatically generated via SurveyCTO programming syntax: 1/2/3- 1 digit for control/intervention1/intervention; then 2 digits unique to each clinic/site; and 3 digits 001-500 for pt). 145001, 238002, 367003 for example. We will retain MSK client ID only to ensure the correct participant ID matches with the MSK client ID so that the correct client is followed-up.
- The MS-Excel file with the key to the code of names associated with their respective codes will be kept electronically and will be encrypted. Only the researchers and assistants associated with this study will have access to this document.
- At the end of every day of data collection, the data will be uploaded to a secure server; survey data will never be stored on the tablet or smart phone. The tablets will be locked and stored in a secure storage unit at IPA-Kenya offices. Prior to being uploaded, the enumerator will retain this data on their password-protected tablet, where only they will have access. The enumerator will not leave the facility (either at the end of the day or any time before) without first uploading any data to the secure, encrypted server and removing the data from their tablet.
- Computers holding subject data will also be password protected and will be stored on a secure online server which is password protected. A back-up drive containing a copy of all the data will be securely kept in a separate location.
  1. **Data Analysis**:

**Qualitative Data Analysis Plan**

We will use descriptive analysis, bivariate analyses, and multivariate analyses to evaluate the intervention and to look for differences within and between groups. We will also look at the association between measures of person-centered care and various health outcomes and behaviors related to post abortion family planning, mental health, and stigma, among others. We will also use longitudinal data analysis methods to look at the follow-up data collected from follow-up surveys to look for associations between health outcomes and different componenets of each internvetion arm compared to control arm and compared to different follow-up points. All data will be analyzed using Stata statistical software.

1. **TIME FRAME/DURATION OF THE PROJECT:**

**Pilot Study:** Approximately 7 months

**Definitive Study:** April – **December** 2018

**Data** **Analysis**: **December 2018** – **March** 2019

**Report Preparation: April – December 2019**

1. **ETHICAL CONSIDERATIONS**:
   1. **Human Subjects:**
2. **“First, do no harm”:** There is minimal risk to study participants. However we recognize that women who have recently had an SA/PAC are vulnerable subjects with the possibility of experiencing health problems while they are participating in data collection activities. Also, some of the evaluation questions in the baseline and follow-up surveys ask about mental health, stigma, and social support which could bring up troubling experiences for some participants. The IPA research assistants will be trained in how to respond compassionately and appropriately. Additionally, we will have a referral procedure in place in the event that any study participant becomes unwell, shares about severe depression, suicidal ideation, and/or rape/incest, and needs to be referred to receive medical care.
3. **Direct Benefit:** This study aims to improve the quality of care for SA/PAC clients. Improving person-centered quality for post abortion services may provide a benefit to women. These benefits may lead to changes in health-seeking behavior or adherence with medical advice which could lead to improved outcomes among women during the study period, or who seek care in the future. These benefits will be distributed evenly across clients of the participating MSK facilities.

   While there are no direct benefits to subjects participating in this research, we anticipate that women who recently underwent an SA/PAC procedure and who participate in the intervention arms of this study will receive emotional and psychological support for their participation. Women may benefit from the opportunity to share their experience in a safe and unified environment. Additionally, for the group speaking with a peer counselor, speaking with someone who has gone through a similar experience may reduce the feeling of stigma attached with their decision to get an abortion, which may increase their sense of self-efficacy and resilience. Women who are in the control arm may not experience the same emotional support as those participating in the intervention arms but they may find it beneficial to to talk about and provide feedback on their experience with an the IPA researcher during baseline and follow-up.

All study participants will receive referrals to or information regarding local health and social service programs, including domestic violence or mental health programs, of which they may have otherwise been unaware.

1. **Informed Consent:** For the surveys, the researcher will obtain and document informed consent (verbally read to respondents and respondents will either sign or mark the page with an “x” or provide a thumb print if they cannot sign their name) and give the participant a copy of the informed consent form. Consent will be obtained by local researcher assistants, hired and supervised by study PIs. The informed consent process will include informing all potential participants of: 1) the study purpose and methods; 2) the study risks and benefits; 3) procedures to protect their privacy and confidentiality; 4) their rights to refuse to answer any question(s) or withdraw from the study at any time; and 5) persons to contact if they have any questions about the study after the completion of the interview/survey.

The interviewer will ask follow-up questions to ensure that the informed consent process has been well understood by the potential participant. If a potential participant agrees to participate, he/she will be given her own copy of the informed consent form outlining his/her rights as a research subject. This page will also include names and phone numbers of persons to contact with any questions regarding the study. We will inform the study participant that having this information sheet could indicate that they have been a part of this study, and therefore they should keep it safe and away from those who they do not wish to see it.

1. **Confidentiality:** For the surveys, in order to ensure women’s privacy and confidentiality, we will recruit women while they are still at the facility. We will work closely with the facilities to identify the appropriate timing and method of approaching women so as to maintain their privacy. To the extent possible, we will try to ensure that providers do not know who is interviewed or surveyed. We will reassure women that all responses will be kept confidential, that the facility will not know what they said, and that we are not affiliated with the facility, etc. The study staff will work closely with facility managers to secure a private room for the screening, informed consent process, and the survey. The study staff will visit the private room prior to conducting the study activities to ensure privacy of the participant.

All electronic data will be stored for the long term in password-protected folders in the secured data servers of UCSF and at our partners’ location. Hard copy data like consent forms will be kept under lock and key with restricted access. Phones to be used to make follow-up calls will only be accessed by study staff.

- 1. **Ethical Review**

This protocol, study tools, and consent forms will be submitted for ethical approval to the independent Ethics Review Committee at Marie Stopes International, the institutional review board at University of California San Francisco, and the local review board, the Kenyan Medical Research Institute.

- 1. **New drugs and/or Procedures (side effects, reactions, etc.):** Not applicable
  2. **Animal Subjects:** Not applicable

1. **EXPECTED APPLICATION OF THE RESULTS:**

The study results (deidentified and aggregated) will be shared with the MSK facilities through quality advisory groups. In addition, findings will be shared with MSI and will be written up in journal articles and shared with the academic community. The results of this study will lead to the potential scale up of this intervention to other regions of Kenya or larger populations. If the scale up of this intervention is found successful we will share our results with Kenya’s Ministry of Health.

1. **REFERENCES:**

Allison, Susannah, Jose A. Bauermeister, Sheana Bull, Marguerita Lightfoot, Brian Mustanski, Ross Shegog, and Deb Levine. 2012. “The Intersection of Youth, Technology, and New Media with Sexual Health: Moving the Research Agenda Forward.” *Journal of Adolescent Health* 51 (3): 207–12. doi:10.1016/j.jadohealth.2012.06.012.

Amirkhanian, Yuri A., Jeffrey A. Kelly, Elena Kabakchieva, Timothy L. McAuliffe, and Sylvia Vassileva. 2003. “Evaluation of a Social Network HIV Prevention Intervention Program for Young Men Who Have Sex with Men in Russia and Bulgaria.” *AIDS Education and Prevention* 15 (3): 205–20. doi:10.1521/aeap.15.4.205.23832.

Brookman-Amissah, Eunice, and Josephine Banda Moyo. 2004. “Abortion Law Reform in Sub-Saharan Africa: No Turning Back.” *Reproductive Health Matters* 12 (24): 227–34. doi:10.1016/S0968-8080(04)24026-5.

Bull, Sheana S., Deborah K. Levine, Sandra R. Black, Sarah J. Schmiege, and John Santelli. 2012. “Social Media-Delivered Sexual Health Intervention: A Cluster Randomized Controlled Trial.” *American Journal of Preventive Medicine* 43 (5): 467–74. doi:10.1016/j.amepre.2012.07.022.

Ceylan, A., Ertem, M., Saka, G., & Akdeniz, N. (2009). Post abortion family planning counseling as a tool to increase contraception use. *BMC Public Health*, *9*, 20. https://doi.org/10.1186/1471-2458-9-20

Charles, V. E., Polis, C. B., Sridhara, S. K., & Blum, R. W. (2008). Abortion and long-term mental health outcomes: a systematic review of the evidence. *Contraception*, *78*(6), 436–450. https://doi.org/10.1016/j.contraception.2008.07.005

Cuijpers, Pim. 2002. “Effective Ingredients of School-Based Drug Prevention Programs. A Systematic Review.” *Addictive Behaviors* 27 (6): 1009–23.

Desai, M., Phillips-Howard, P. A., Odhiambo, F. O., Katana, A., Ouma, P., Hamel, M. J., … Laserson, K. F. (2013). An analysis of pregnancy-related mortality in the KEMRI/CDC health and demographic surveillance system in western Kenya. *PloS One*, *8*(7), e68733. https://doi.org/10.1371/journal.pone.0068733

Evens, Emily, Rose Otieno-Masaba, Margaret Eicheleay, Donna McCarraher, Gwyn Hainsworth, Cate Lane, Margaret Makumi, and Pamela Onduso. 2013. “Post-Abortion Care Services for Youth and Adult Clients in Kenya: A Comparison of Services, Client Satisfaction and Provider Attitudes.” *Journal of Biosocial Science*, 1–15. doi:10.1017/S0021932013000230.

Guest, G., Namey, E., & McKenna, K. (2016) How Many Focus Groups are Enough? Building an Evidence Base for Nonprobability Sample Size. *Field Methods*, 29(1). 3-22. http://journals.sagepub.com/doi/10.1177/1525822X16639015

Harris, G. E., & Larsen, D. (2007). HIV Peer Counseling and the Development of Hope: Perspectives from Peer Counselors and Peer Counseling Recipients. *AIDS Patient Care and STDs*, *21*(11), 843–860. https://doi.org/10.1089/apc.2006.0207

Ikamari, Lawrence, Chimaraoke Izugbara, and Rhoune Ochako. 2013. “Prevalence and Determinants of Unintended Pregnancy among Women in Nairobi, Kenya.” *BMC Pregnancy and Childbirth* 13 (1). BMC Pregnancy and Childbirth: 1–9. doi:10.1186/1471-2393-13-69.

Kelly, Jeffrey A, Debra A Murphy, Kathleen J Sikkema, Timothy L McAuliffe, Roger A Roffman, Laura J Solomon, Richard A Winett, and Seth C Kalichman. 1997. “Randomised, Controlled, Community-Level HIV-Prevention Intervention for Sexual-Risk Behaviour among Homosexual Men in US Cities.” *The Lancet* 350 (9090): 1500–1505. doi:10.1016/S0140-6736(97)07439-4.

Latkin, Carl A., Susan Sherman, and Amy Knowlton. 2003. “HIV Prevention among Drug Users: Outcome of a Network-Oriented Peer Outreach Intervention.” *Health Psychology* 22 (4): 332–39. doi:10.1037/0278-6133.22.4.332.

Levine, Deb. 2011. “Using Technology, New Media, and Mobile for Sexual and Reproductive Health.” *Sexuality Research and Social Policy* 8 (1): 18–26. doi:10.1007/s13178-011-0040-7.

Lightfoot, Marguerita, W. Scott Comulada, and Gabriel Stover. 2007. “Computerized HIV Preventive Intervention for Adolescents: Indications of Efficacy.” *American Journal of Public Health* 97 (6): 1027–30. doi:10.2105/AJPH.2005.072652.

Magadi, Monica Akinyi. 2003. “Unplanned Childbearing in Kenya: The Socio-Demographic Correlates and the Extent of Repeatability among Women.” *Social Science and Medicine* 56 (1): 167–78. doi:10.1016/S0277-9536(02)00018-7.

Marie Stopes International (MSK). 2016. “Marie Stopes Kenya.” Accessed February 15. https://mariestopes.org/where-in-the-world#kenya.

McLaughlin, Margaret, Yujung Nam, Jessica Gould, Courtney Pade, Kathleen A. Meeske, Kathleen S. Ruccione, and Janet Fulk. 2012. “A Videosharing Social Networking Intervention for Young Adult Cancer Survivors.” *Computers in Human Behavior* 28 (2): 631–41. doi:10.1016/j.chb.2011.11.009.

Mellanby, A. R., R. G. Newcombe, J. Rees, and J. H. Tripp. 2001. “A Comparative Study of Peer-Led and Adult-Led School Sex Education.” *Health Education Research* 16 (4): 481–92.

Mohamed, Shukri F, Chimaraoke Izugbara, Ann M Moore, Michael Mutua, Elizabeth W Kimani-Murage, Abdhalah K Ziraba, Akinrinola Bankole, Susheela D Singh, and Caroline Egesa. 2015. “The Estimated Incidence of Induced Abortion in Kenya: A Cross-Sectional Study.” *BMC Pregnancy and Childbirth* 15 (January): 185. doi:10.1186/s12884-015-0621-1.

Oyieke, J. B. O., Obore, S., & Kigondu, C. S. (2006). Millennium development goal 5: a review of maternal mortality at the Kenyatta National Hospital, Nairobi. *East African Medical Journal*, *83*(1), 4–9.

Pulerwitz, Julie, Annie Michaelis, Ravi Verma, and Ellen Weiss. 2010. “Addressing Gender Dynamics and Engaging Men in HIV Programs: Lessons Learned from Horizons Research.” *Public Health Reports* 125 (2): 282–92.

Republic of Kenya. (2010). *The Constitution of Kenya, 2010*. Nairobi: Republic of Kenya.

Rosenstock, I. M., Strecher, V. J., & Becker, M. H. (1988). Social learning theory and the health belief model. *Health Education & Behavior*, *15*(2), 175–183.

Steinberg, J. R., Tschann, J. M., Furgerson, D., & Harper, C. C. (2016). Psychosocial factors and pre-abortion psychological health: The significance of stigma. *Social Science & Medicine (1982)*, *150*, 67–75. https://doi.org/10.1016/j.socscimed.2015.12.007

Teti, M., Rubinstein, S., Lloyd, L., Aaron, E., Merron-Brainerd, J., Spencer, S., … Gold, M. (2007). The Protect and Respect Program: A Sexual Risk Reduction Intervention for Women Living with HIV/AIDS. *AIDS and Behavior*, *11*(1), 106–116. https://doi.org/10.1007/s10461-007-9275-y

Turner, G., and J. Shepherd. 1999. “A Method in Search of a Theory: Peer Education and Health Promotion.” *Health Education Research* 14 (2): 235–47. doi:10.1093/her/14.2.235.

UCSF, IPA, & MSK. (n.d.). *Characterizing the Perceptions and Experiences of Post-Abortion Care (PAC) among young Kenyan Women in Nairobi*. University of California, San Francisco.

Ulin, P. R., Robinson, E. T., & Tolley, E. E. (2004). Qualitative Methods in Public Health: A Field Guide for Applied Research. John Wiley & Sons.

Upadhyay, U. D., Cockrill, K., & Freedman, L. R. (2010). Informing abortion counseling: An examination of evidence-based practices used in emotional care for other stigmatized and sensitive health issues. *Patient Education and Counseling*, *81*(3), 415–421. https://doi.org/10.1016/j.pec.2010.08.026

Wood, J. V. (1989). Theory and research concerning social comparisons of personal attributes. *Psychological Bulletin*, *106*(2), 231.

World Health Organization. (2011). *Unsafe abortion: global and regional estimates of the incidence of unsafe abortion and associated mortality in 2008. -- 6th ed.* WHO.

World Health Organization (WHO). (2012). *Safe Abortion: Technical and Policy Guidance for Health Systems*. Geneva: WHO.

Yegon, E. K., Kabanya, P. M., Echoka, E., & Osur, J. (2016). Understanding abortion-related stigma and incidence of unsafe abortion: experiences from community members in Machakos and Trans Nzoia counties Kenya. *The Pan African Medical Journal*, *24*, 258. https://doi.org/10.11604/pamj.2016.24.258.7567

Ziraba, A. K., Madise, N., Mills, S., Kyobutungi, C., & Ezeh, A. (2009). Maternal mortality in the informal settlements of Nairobi city: what do we know? *Reproductive Health*, *6*, 6. https://doi.org/10.1186/1742-4755-6-6

The magnitude of abortion complications in Kenya

Hailemichael Gebreselassie,

a

Maria F. Gallo,

b

Anthony Monyo,

a

Brooke R. Johnson

The magnitude of abortion complications in Kenya

Hailemichael Gebreselassie,

a

Maria F. Gallo,

b

Anthony Monyo,

a

Brooke R. Johnson

The magnitude of abortion complications in Kenya

Hailemichael Gebreselassie,

a

Maria F. Gallo,

b

Anthony Monyo,

a

Brooke R. Johnson
